# Correlation between community balance and mobility scale (CB&M) with a battery of outcome measures to assess balance in Parkinson’s disease – a cross-sectional study

**DOI:** 10.1186/s40945-021-00117-y

**Published:** 2021-11-08

**Authors:** Ziona Lionel Dsouza, Sydney Roshan Rebello, Cherishma Dsilva

**Affiliations:** 1 Department of Physiotherapy, Ramaiah Medical College Hospital, M S Ramaiah nagar, MSRIT Post, Bangalore, 560054 India; 2 Department of Physiotherapy, Father Muller College of Allied Health Sciences, Mangalore, 575002 India

**Keywords:** Community Balance & Mobility, Parkinson, Balance, Berg balance scale, Functional reach test, Timed UP & GO

## Abstract

**Background:**

Evaluating balance in a functional context that integrates challenging tasks frequently performed in the community is essential to identify community-dwelling individuals who are at risk of falls in early Parkinson Disease (PD) than a simple balance measure. Community Balance and Mobility (CB&M) scale is one such measure that evaluates severe deficits in gait, balance, and mobility. The risk of falling and fear of fall is common among PD individuals and this affects the day to day functioning as well as the quality of life. Early identification of individuals who may be at risk to fall will lead to intervention strategies that can help to with balance issues. The aim of this study was to correlate between Community Balance and Mobility with a battery of outcome measures commonly used to assess balance in Parkinson’s disease.

**Methods:**

A cross sectional study design; with individuals referred to Outpatient physiotherapy department, diagnosed with idiopathic Parkinson’s disease, independently mobile and on a stable drug regimen referred by the neurologist; were screened and recruited by convenience sampling. With written informed consent, demographic data gathered and scales such as Berg Balance scale, Community balance & mobility scale, Functional Reach test and Timed up and go test were administered with an ample amount of rest.

**Results:**

The results obtained were documented and analysed using Karl Pearson’s correlation coefficient. Significant correlation between CB&M and BBS (r = 0.795) was found, CB&M and TUG (r = − 0.755), CB&M and FRT (r = 0.772).

**Conclusion:**

CB&M is a useful measure which integrates items that challenge balance in the community context. It has been used to assess high functioning community dwelling individuals and hence may be apt for individuals with early Parkinson’s, since the tasks to be performed in CB&M are challenging and these simulate community level activities where the risk of falls is higher. It may well be a good tool to assess early Parkinson’s; their level of balance, community level activity and without need for sophisticated & expensive equipment.

## Background

Postural instability and balance deficits due to abnormality in postural adjustments, truncal and extremity rigidity, loss of postural reflexes, motor incoordination, and akinesis are the most devastating impairments and predispose the patient to unexpected falls, thereby increasing the risk of mortality and morbidity among patients with Parkinson disease [1, 2].

Evidence suggests that physical therapy interventions improve balance deficits in PD thereby minimizing disability, maximizing independent living and mobility [3]. Careful evaluation of balance is essential with mild impairments in the early PD since it enables the Physical therapists to determine the degree to which they need to address fall prevention and provide treatment strategies tailored to specific contributing factors. Simple tests such as timed single-limb stance test (eyes open and closed), Romberg test, and modified sit-reach test do not provide adequate information regarding the complex balance activity [4]. Commonly used measures for balance are Berg Balance Scale (BBS) [5], Timed Up and Go test (TUG) [6, 7], Functional Reach Test (FRT) [8], Tinetti Performance Oriented Mobility Assessment (POMA) [9], Balance Evaluation Systems Test, and Mini Balance Evaluation Systems Test (BESTest and MiniBESTest) [10].

Evaluating balance in a functional context that integrates challenging tasks frequently carried out in the community is essential to identify community-dwelling individuals who are at risk of falls in early PD than any simple balance measure. Various aspects of posture and movement are included in the CB&M which represent motor skills necessary to perform the function in the community like walking and looking, lateral foot scooting, running with controlled steps, forward to backward walking, hopping forward, and crouch and walk are assessed. Community Balance and Mobility (CB&M) scale is one such measure that evaluates severe deficits in gait, balance, and mobility while its psychometric properties have been established in other populations (stroke, community-dwelling older adults, TBI) [11]. Susceptibility has been reported in BBS when compared to CB&M [12]. However, despite the usefulness of this scale, literature shows that no studies have been conducted on Parkinson’s disease patients. This study aimed to correlate Community Balance and Mobility scale with a battery of outcomes commonly used to assess balance among community-residing individuals with Parkinson’s disease.

## Methods

This study employed a cross-sectional study design. The population for this study was referred by the consultant neurologist in an outpatient setting with a confirmed diagnosis of PD. The ethics committee approval was obtained before patients were recruited for the study and all participants were ambulatory without any assistive device or physical assistance. A convenient sampling technique was used. Subjects were assessed as and when they were referred by the Neurologist across the day. After screening fifty-six patients, nine were excluded and forty-seven met the inclusion criteria were assessed.

### Participants

Inclusion criteria for participation were, confirmed diagnosis of idiopathic PD, disease severity on Hoehn and Yahr (H&Y) Stage was 2 and 3. The exclusion criteria were secondary Parkinsonism, disorders that affect gait (eg, peripheral neuropathy, orthopedic injuries), and impaired cognitive function (Mini-mental status examination less than 24 scores). All forty-seven participants were given verbal and written information with written informed consent obtained before participation.

### Assessment of balance and functional mobility

Subject’s demographic data and following outcome measures: Community Balance and Mobility scale, Berg Balance Scale, Timed Up and Go test, Functional Reach Test were administered for all the participants.

#### Community balance and mobility scale

The Community Balance and Mobility Scale (CB&M) Scoring is based on a scale of 0 to 5, with a score of 0 reflecting complete inability to perform the task and a score of 5 reflecting the most successful completion of the task possible with an exception of one item i.e. descending stairs which is scored from 0 to 6. Scores range from 0 to 96 and 13 items were scored upon completion of the first trial of each item. Higher scores represent better balance and mobility [12]. The validity and reliability of this outcome measure has been established for persons with traumatic brain injury but not for patients with Parkinson’s [13].

#### Berg balance scale

The BBS assesses functional balance on a scale of 0 to 4 for 14 tasks with 0 represents inability to perform while 4 indicates able to perform independently. Approximately 20 min is required to complete the scale and the maximum score of 56 is indicative of better balance. Qutubuddin AA et al. have also concluded that BBS is valid to measure balance not only as a screening tool but also as an ongoing assessment for patients undergoing intervention [5].

#### Timed up & go

The ability to rise from a seated position, walk 3 m, turn and walk back to the starting position and sit down is timed on the Timed Up and Go (TUG) test [6]. A recent systematic review by Mollinedo I et al. concluded that the use of Timed Up and Go has good reliability and validity in Parkinson disease patients [7].

#### Functional reach test

The Functional Reach Test was performed in standing next to a wall but without touching it with shoulder-held in 90 degrees, the starting position at the 3rd metacarpal head on the yardstick. The patients reach as far as possible without taking a step. The difference between the start and end position determines the distance covered, measured in centimeters. The average of the last two out of the three was noted [8]. An ample amount of breaks were provided between the task and the outcomes were administered in the same series for all the included participants and the obtained results were documented and analyzed.

#### Statistical analysis

The CB&M, BBS, TUG, and FRT assessments were scored according to the test instructions, and a summary score was derived for each. Data were entered into SPSS for Windows (version 23). Scatterplots were drawn to examine the relationship between CB&M, BBS, TUG, and FRT. A Shapiro-Wilk test (*p* > .05) and visual inspection of their histograms, normal Q –Q plots, and box plots showed that the scores were approximately normally distributed for stage 2 &3 Hoehn & Yahr with the skewness of -. 149 (SE = .393) and a Kurtosis of – .981 (SE = .768) for the subjects in Stage 2 and a skewness of .760 (SE = .661) and a kurtosis of .443 (SE = 1.279) for subjects in Stage 3. Bivariate correlation among all the measures was calculated; with the degree of relation between the indicators assessed by using Karl Pearson’s correlation coefficient (r). The floor and ceiling effects were calculated as the percentage of the sample scoring the minimum or maximum possible scores, respectively. The ceiling effect was defined only in the clinical assessment that had a clear maximum score of 96 on CB&M and 56 on BBS. Sub-group correlation analysis of CB&M, BBS, TUG, and FRT in stages 2 & 3 during ON and OFF medication was carried out using Spearman Correlation Coefficient (rho). All probability (p) values in this study were calculated within a confidence interval of 95%. The significance was set up at p>/=0.05. Fallers and Non-fallers.

## Results

Table 1 displays the demographic and clinical characteristics of the participants. Forty-seven participants (30 men, 17 women), aged 36 to 83 years were assessed. About 36 patients were in Stage 2 and 11 patients belonged to Stage 3 according to Hoehn and Yahr staging. Twenty-seven patients were on OFF medication during assessment and 22 were ON medication.

**Table 1:** Demographic and clinical characteristics of the participants

Variables	Mean ± SD		
Age of the patient (years)	62.61 ± 9.76		
Duration of diseases (years)	2.63 ± 2.35		
Staging	2	36 patients	
	3	11 patients	
On phase	20 (42.6%)		16 (Stage 2)
			4 (Stage 3)
Off phase	27 (57.4%)		20 (Stage 2)
			7 (Stage 3)

Table 2 Describes the clinical data of the subjects. The average CB&M score for all the subjects was 39.38 ± 22.48 with a minimum score of 4 and a maximum score of 85 out of 96. The average BBS scores were 42.66 ± 11.43 with subjects scoring between 16 to the maximum score. The average TUG scores were 14.05 ± 6.06 in seconds. The average FRT scores were 19.91 ± 6.83 in centimeters.

**Table 2:** Clinical data of the subjects

	N	Minimum	Maximum	Mean	Standard deviation
**CB&M**	47	4	85	39.38	22.48
**BBS**	47	16	56	42.66	11.433
**TUG**	47	5.7	29.9	14.051	6.0639
**FRT**	47	5	32	19.91	6.830

**Fig. 1:**
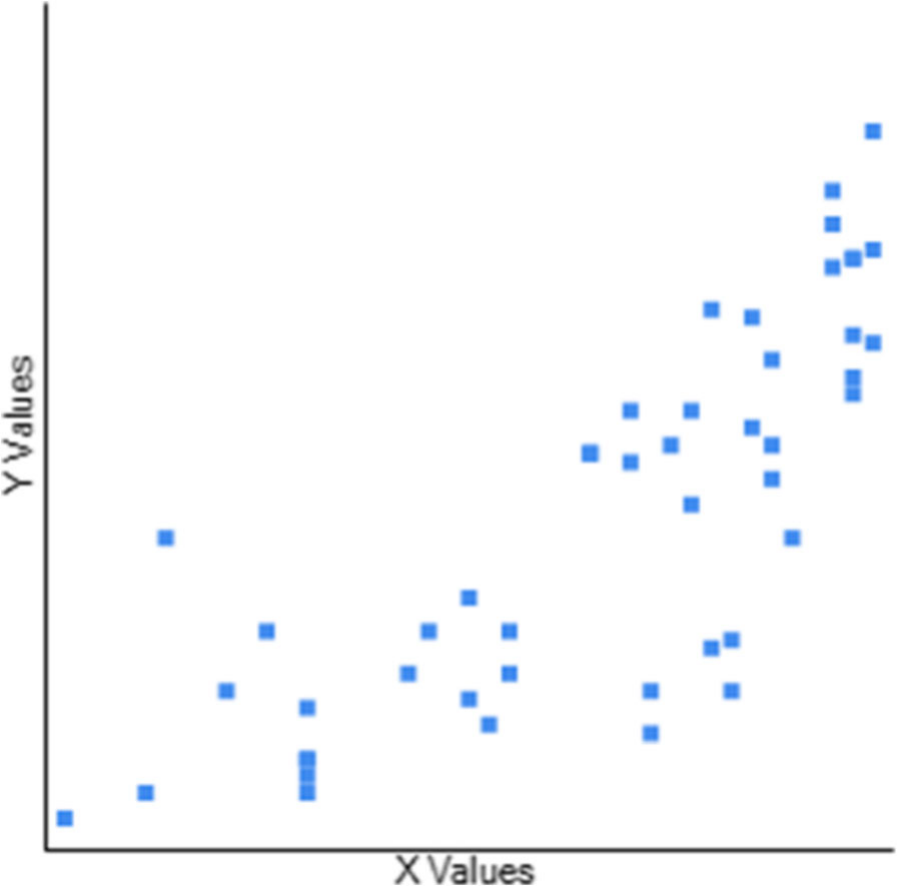
Graphical representation of correlation between Community Balance and Mobility scale and Berg Balance Scale. Y axis: Community Balance and Mobility scale. X axis: Berg Balance Scale. r = 0.7948

**Fig. 2:**
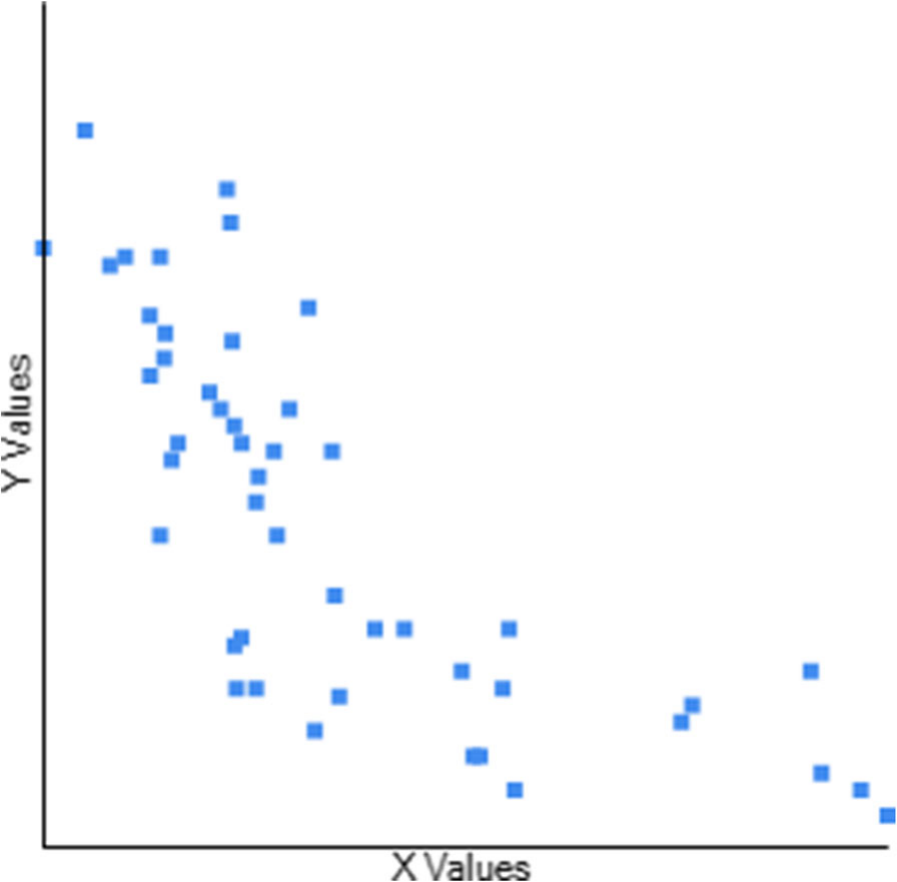
Graphical representation of correlation between Community Balance and Mobility and Timed Up and Go testY axis: Community Balance and Mobility scale. X axis: Timed Up & Go test. r = -0.7552

**Fig. 3:**
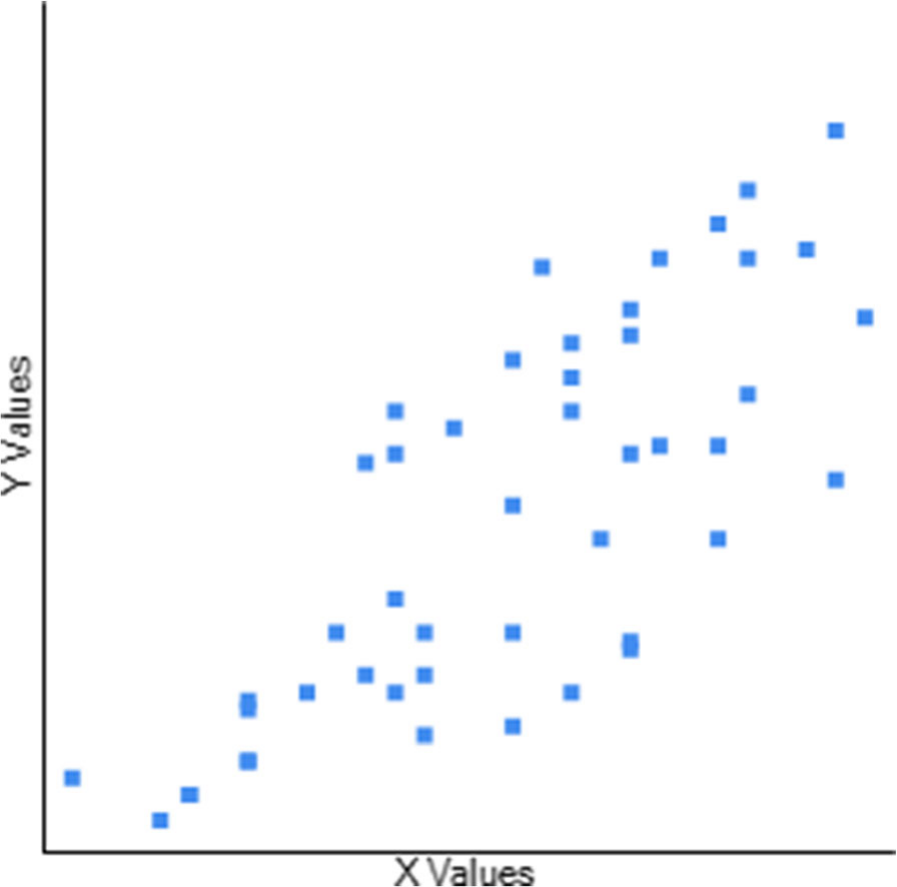
Graphical representation of correlation between Community Balance and Mobility and Functional Reach Test. Y axis: Community Balance and Mobility scale. X axis: Functional Reach test. r = 0.7715

Table 3 Describes the Concurrent Validity of CB&M to BBS, TUG, and FRT. The scores of 47 participants were used to determine the association between the CB&M and BBS, TUG, and FRT. CB&M showed a high positive correlation (r = 0.795) to BBS (Fig. 1), suggesting that higher performance on BBS is related to higher CB&M scores and vice-versa. Notably, higher ratings on CB&M suggest that patients have a “high level” balance and mobility and a lower risk of falls. CB&M showed a high negative correlation (r = − 0.755) to TUG suggesting that higher scores on the CB&M are related to lower TUG scores indicating high-level balance and mobility in the community and have excellent functional performance (Fig. 2). CB&M showed a high positive correlation (r = 0.772) to FRT suggestive of that a higher score on CB&M is related to higher scores on FRT (Fig. 3) (Raithel,2008). Table 4 demonstrates the ceiling effects of CB&M and BBS in subjects with PD. Table 5 shows sub-group analysis of each scale in Stages 2 & 3 according to participants assessed while ON and OFF medication.

**Table 3:** Correlation of CB&M to BBS, TUG, and FRT

	BBS	TUG	FRT
**CB&M**	O.795	−0.755	0.772
**P**	< .001	< .001	< .001

**Table 4:** Shows BBS has a ceiling effect while compared to CB&M in subjects with PD

	Mean ± SD	Minimum score	Maximum score	Ceiling effect
**CB&M**	**39.38 ± 22.48**	**4**	**85**	**0%**
**BBS**	**42.66 ± 11.43**	**16**	**56**	**14.89%**

**Table 5:** Shows sub-group analysis of each scale in Stages 2 & 3 according to participants assessed while ON and OFF medication

	ON Medication		OFF Medication	
	Mean (SD)	95% CI	Mean (SD)	95% CI
**CB&M Stage 2**	46.13 (28.87)	34.47–57.78	46.60 (19.97)	37.25–55.95
**CB&M Stage 3**	20.00 (12.27)	0.47–39.53	14.43 (8.40)	6.66–22.20
**BBS Stage 2**	45.38 (11.15)	39.43–51.32	46.45 (8.50)	42.47–50.43
**BBS Stage 3**	35.25 (11.41)	17.09–53.41	29.86 (10.07)	20.54–39.17
**TUG Stage 2**	11.24 (3.22)	9.52–12.96	12.96 (5.16)	10.54–15.38
**TUG Stage 3**	15.77 (4.46)	8.66–22.87	22.59 (7.01)	16.11–29.08
**FRT Stage 2**	22.25 (6.40)	18.84–25.66	20.90 (6.38)	17.91–23.89
**FRT Stage 3**	16.00 (7.57)	3.95–28.05	12.43 (4.07)	8.66–16.20

Table 6, 7, 8, 9 Displays correlation between CB&M, BBS, TUG & FRT using Spearman’s correlation coefficient among Stage 2 & 3 (ON & OFF medication). Table 10 displays no of Fallers and Non-fallers in Stage 2 & 3.

**Table 6:** Stage 2 ON medication

	BBS	TUG	FRT
**CB&M**	.773	−.853	.732
**p**	<. 001	<. 001	. 001

**Table 7:** Stage 2 OFF medication

	BBS	TUG	FRT
**CB&M**	.737	−.718	.609
**p**	<.001	<.001	.004

**Table 8:** Stage 3 ON medication

	BBS	TUG	FRT
**CB&M**	1.00	−.949	1.0
**p**		**.051**

**Table 9:** Stage 3 OFF medication

	BBS	TUG	FRT
**CB&M**	.827	−.631	.991
**p**	.022	1.29	<.001

**Table 10:** Fallers and Non-Fallers according to Stage 2 & 3

	No. of Non Fallers (***n*** = 33)	No. of Fallers (***n*** = 14)
**Stage 2**	31	5 (13.88%)
**Stage 3**	2	9 (81.82%)

## Discussion

The purpose of this study was to correlate CB&M with other commonly used balance outcome measures in evaluating functional balance and mobility in patients with Parkinson’s disease. The Community Balance & Mobility scale has been validated and found to be reliable among stroke, older adults, and traumatic brain injury [11–13]. It is a scale constructed to challenge the individual’s ability to perform the activities and to detect persistent balance dysfunction along with mobility during the activities. It incorporates several demanding tasks that are commonly performed in the community environment which involves dual tasking.

The findings from this study show that the CB&M scale has an excellent correlation with other performance-based measures we used on Parkinson’s patients. Intact balance is integral for all functional activities, right from upright sitting to standing and for ambulation. Patients with Parkinson’s disease who have impaired balance are at high risk of falls and those with a history of falls require appropriate intervention [12]. Forty-seven (*n* = 47) Community-dwelling individuals diagnosed with Parkinson were recruited for this study and most were above the age of 60 belonging either to Stage 2 or 3 on Hoehn and Yahr scale.

### Correlation of CB&M versus berg balance scale, TUG and FRT

CB&M showed a strong correlation with BBS, TUG and FRT (r = 0.79, r = − 0.75, r = 0.77) respectively (Table 3). All the scales in this study are commonly used to measure balance, BBS measures static and dynamic balance, TUG assesses transfers and mobility while FRT measures anteroposterior stability. Despite the results demonstrated a strong correlation among all, participants managed to achieve scores that did not indicate any balance impairment while scores on CB&M continued to remain lower.

The results obtained in our study demonstrate that those approaching the maximal possible score of 56 on the BBS and the TUG scores at or below 12.3 s and the FRT score of above 25 cm, attained a wide range of scores 60–85 out of 96 on the CB&M. This may be due to the complexity of tasks incorporated in the CB&M measure. CB&M needs dual-tasking in most of the items on the scale. For example, CB&M includes an item walking and looking at a target point. Most patients found it to be demanding tasks to continuing to walk in a straight path while gazing at the objects in the environment. This could be due to attention allocation which may drastically change balance while dual-tasking if a cognitive or motor component is added to a single task.

The items on CB&M are more challenging even for healthy community-dwelling individuals. The reason being, CB&M measures the underlying mechanism of postural control in different tasks which simulate real-life activities, and measures of static & dynamic balance may not effectively evaluate the same. These findings are in conjunction with a previous study by Knorr S et al. that the highest score attained on the CB&M by the moderate to high functioning stroke participants was comparatively lower than BBS suggesting that this scale provides an adequate level of difficulty to detect balance deficits [11]. Hence balance should be evaluated during mobility tasks making it more applicable to real-life community situations.

### Floor & ceiling effect of CB&M in Parkinson

Most of the individual scores clustered around the maximum possible score on BBS, which suggests that BBS has a ceiling effect that supports the findings of other studies [14]. On the contrary, the current study shows no ceiling or flooring effect for CB&M which is in concurrence with previous studies. Thus, a combination of BBS, TUG, and FRT can be used to evaluate balance but does not challenge balance sufficiently enough to allow the detection of balance impairments early in the disease.

### ON and OFF medication (stage 2 & 3)

A moderate to high correlation (*p* < .001) was observed between CB&M and BBS, TUG and FRT Stage 2 (ON & OFF). Mean scores of CB&M and BBS among Stage 2 participants were similar in both ON and OFF medication groups but subjects achieved scores on the Berg Balance Scale which classify them in the low risk of fall category in comparison to scores achieved on CB&M. Although at present there are no studies on CB&M cut-off scores in PD, lesser scores are suggestive of poor balance and mobility [12]. Even though H & Y Stage 2 categorizes individuals as without impaired balance, our study shows that these individuals may be at risk of falls since scores on the CB&M scale were less while performance on BBS showed lower risk of falls. No significant difference was observed in the mean scores between TUG and FRT. Participants in the study achieved scores of < 16 s on TUG suggestive of low risk of fall and the distance covered on FRT was less than the cut–off score (25.4 cm) indicative of risk of falls. The use of CB&M in Stage 2 may help clinicians to identify possible early risk of falls in comparison to commonly used balance assessments like BBS, TUG, and FRT.

A very low correlation was observed in our study in Stage 3 and this may be due to the number of participants (*n* = 11) being fewer in comparison to Stage 2 (*n* = 36). They also showed a less significant difference in their mean scores on CB&M, much lower than those in Stage 2. The scores on BBS could be categorized under the moderate risk of fall category. Time taken by individuals to complete the TUG was more than the cut – off (< 16 s) in the ON medication group while that of the OFF medication group (Stage 3) was considerably suggestive of increased risk for falls. The distance achieved on the FRT was far less in both the ON and OFF medication group again indicating increased fall risk. Subjects performed poorly in all the balance scales and maybe in Stage 3 of PD, balance impairment is markedly present even though the individual is physically independent. We observed that performance on all the balance outcomes was positively influenced by medication [15] and future studies may also consider recruiting subjects to test and re-test, when ON and OFF medication. This would help in identifying PD patients with unnoticeable balance impairments and identify early risk of fall than merely waiting for the progression of the disease to result in falls.

#### Fallers and non-fallers

In our study, a small percentage of participants had a history of falls as early as Stage 2. It has been observed that subtle impairment of balance may be masked as they may not be visually obvious, while the use of medication may further go on to conceal the balance issues and the H &Y scale describes the severity of PD based on clinical observation of an individual’s presenting symptoms [16]. Therefore, it is essential to carry out balance assessments during the “off” phase of medication giving a clear picture of balance issues. It is also prudent to screen during the “on” phase because patients typically ambulate and are predisposed to fall.

## Conclusion

Community Balance and Mobility is a scale that can be used to evaluate balance in Parkinson’s disease. It is straightforward to carry out, requires least of equipment, and integrates tasks that challenge balance in the community context beyond conventional measures like BBS, TUG, and FRT. There was no ceiling or flooring effect seen for CB&M in the current study while a ceiling effect was observed for BBS when compared to CB&M. BBS, TUG, and FRT are commonly used but may not challenge balance adequately to allow the detection of balance impairments in the early stage of PD. Hence, CB&M may be a appropriate measure for persons with PD. Future studies with larger sample size, also taking into account the number of falls, fear of falls, and participants could be evaluated during the OFF and ON phase of medication.

## Data Availability

The datasets used and/or analysed during the study are available from the corresponding author on reasonable request.

## Abbreviations

PD: Parkinson’s disease
CB&M: Community Balance and Mobility
BBS: Berg Balance Scale
TUG: Timed Up and Go
FRT: Functional Reach Test
BESTest: Balance Evaluation System Test
UPDRS: Unified Parkinson Disease Rating Scale
H&Y: Hoehn & Yahr
MMSE: Mini-Mental Scale Examination
COG: Center of Gravity
BOS: Base of Support
BD: Balance dysfunction
ADL: Activities of Daily Living
SPSS: Statistical Package for Social Science
